# Red Ear Syndrome: A Case Report and Review of Literature

**DOI:** 10.1155/crot/2549709

**Published:** 2026-06-25

**Authors:** Zaki Alwatban, Wasan Alharbi, Muneera Al Khalifa, Mohammed Al Shehabi

**Affiliations:** ^1^ Ministry of Health, Riyadh, Saudi Arabia, moh.gov.sa; ^2^ Otorhinolaryngology, Head and Neck Surgery Department, Royal Medical Service, Manama, Bahrain

**Keywords:** erythema, erythromelalgia, headache, migraine, red ear syndrome

## Abstract

Red ear syndrome (RES) is a rare disorder characterized by episodic erythema, warmth, and burning pain of the external ear. RES is frequently associated with migraine. We report the case of a 35‐year‐old male with a long‐standing history of poorly controlled migraine who presented with recurrent bilateral auricular erythema and burning pain, predominantly affecting the left ear. Symptoms were triggered by migraine attacks, heat exposure, and ear manipulation and relieved by cooling measures. Clinical examination, laboratory investigations, and imaging were normal. RES was diagnosed after excluding infectious and inflammatory causes. Treatment with indomethacin and magnesium resulted in complete symptom resolution. RES should be considered in patients with recurrent auricular erythema and burning pain, particularly in those with migraine. Careful clinical assessment and systematic exclusion of alternative diagnoses are crucial for identifying RES and preventing inappropriate management.

## 1. Introduction

Red ear syndrome (RES), also known as auricular erythermalgia, is a rare clinical condition first described by Lance in 1994 and further characterized in 1996 [1–4]. RES is defined by paroxysmal episodes of unilateral or bilateral erythema of the external ear, accompanied by warmth and burning pain [1]. These episodes are typically precipitated by mechanical or physiological stimuli, including touching or rubbing the ear, heat exposure, migraine attacks, neck movement, chewing or jaw motion, physical exertion, and emotional stress [4, 5].

The true incidence and prevalence of RES remain unknown, largely owing to the limited number of reported cases; thus, the condition is regarded as rare [4]. The pathophysiology of RES is not fully understood; however, it is generally classified into primary (central) and secondary (peripheral) diseases [6], with some cases demonstrating overlapping features of both mechanisms [7]. Primary RES is typically idiopathic or associated with migraine and trigeminal autonomic cephalalgias (TACs) [5]. In contrast, secondary RES has been associated with upper cervical spine pathology and temporomandibular joint (TMJ) dysfunction [5].

In this study, we present a case of RES in a patient with migraine, accompanied by a comprehensive review of current literature, with particular emphasis on clinical features, proposed pathophysiological mechanisms, differential diagnosis, and management strategies.

## 2. Case Presentation

A 35‐year‐old male with a long‐standing history of poorly controlled migraine since childhood presented to the otorhinolaryngology clinic with recurrent episodes of bilateral ear redness associated with pain. The patient had previously attended multiple clinics and had been repeatedly treated for otitis externa without clinical improvement.

At presentation, he described episodic redness of both ears, predominantly involving the left ear, accompanied by sensations of warmth, burning, and throbbing pain. Similar but milder symptoms were occasionally noted in the right ear. These episodes were temporally associated with unilateral left temporal headaches consistent with his known migraine and were accompanied by visual aura. Symptoms were intermittent, self‐limiting, and recurrent.

Exacerbating factors included migraine attacks, exposure to hot weather, and ear rubbing, whereas symptom relief was achieved with cooling measures and improvement in headache. The patient denied hearing loss, tinnitus, otorrhea, TMJ dysfunction, dental problems, neck pain, rheumatologic or joint symptoms, and nasal or allergic complaints. The patient reported a positive family history of migraine in his father.

On physical examination, bilateral erythema of the auricles with increased warmth was observed, without tenderness or fluctuation (Figure 1). Otoscopic examination revealed normal external auditory canals and intact tympanic membranes bilaterally. The remainder of the otorhinolaryngologic examination, including head and neck assessment and TMJ evaluation, was unremarkable (Figure 2).

**FIGURE 1:**
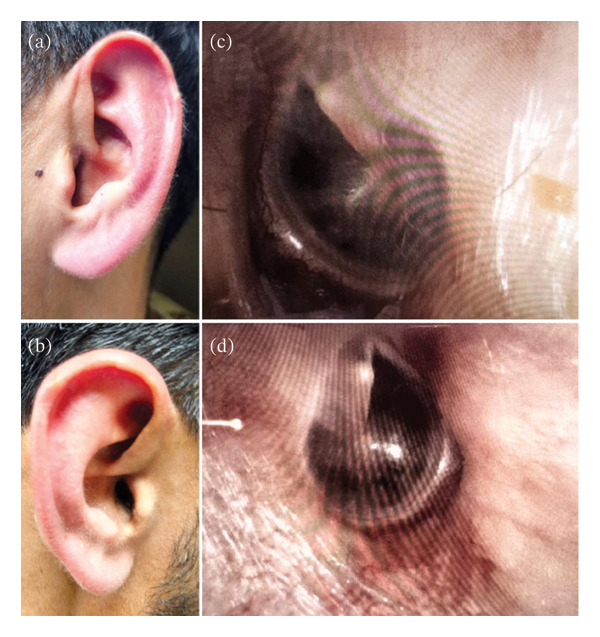
Clinical and otoscopic findings of the patient with red ear syndrome. (a) Left auricle showing erythema and redness. (b) Right auricle showing erythema and redness. (c) Normal otoscopic examination of the left ear. (d) Normal otoscopic examination of the right ear.

**FIGURE 2:**
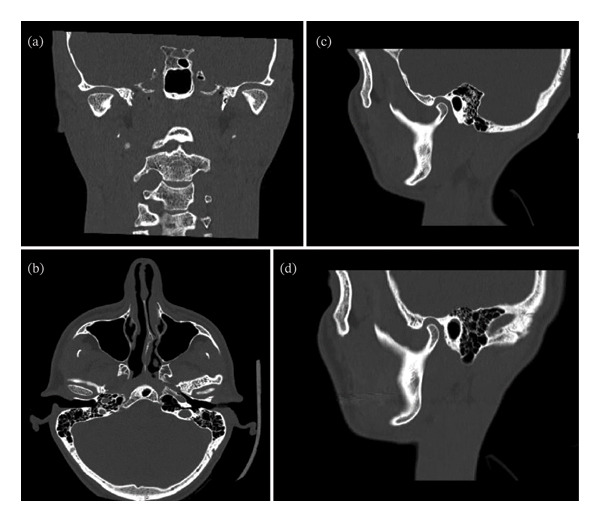
CT scan of the TMJ (multiple views). (a) Coronal CT scan of the TMJ showing a normal TMJ. (b) Axial CT scan of the TMJ showing a normal TMJ. (c) Sagittal CT scan of the right TMJ showing a normal TMJ. (d) Sagittal CT scan of the left TMJ showing a normal TMJ. TMJ, temporomandibular joint; CT, computed tomography.

Laboratory investigations, including routine blood tests and a rheumatologic workup for systemic lupus erythematosus, Sjögren’s syndrome, and relapsing polychondritis, were within normal limits. Computed tomography of the TMJs showed no abnormalities. After excluding alternative diagnoses such as otitis externa, erysipelas, eczematous dermatologic conditions, and relapsing polychondritis, a diagnosis of RES was established.

The patient was treated with indomethacin (25 mg once daily for 3 weeks) and magnesium supplementation (600 mg once daily) and was advised to avoid known triggering factors. He reported marked clinical improvement at 1‐month follow‐up, with complete resolution of ear redness, warmth, and burning sensations (Figure 3). No adverse or unanticipated events were reported.

**FIGURE 3:**
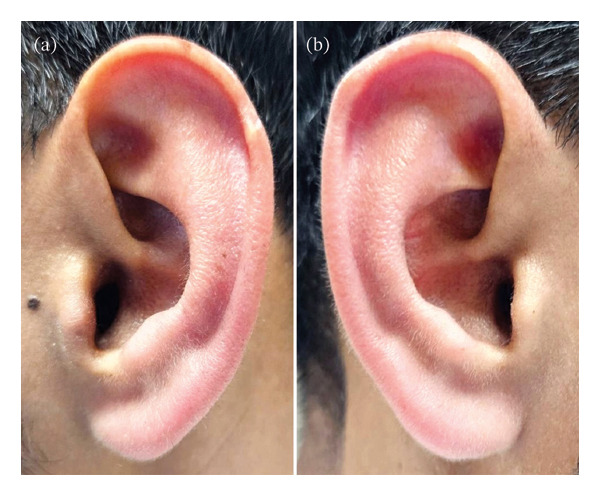
Clinical examination of ear after medical management. (a) Left auricle shows resolution of erythema and redness after medical management. (b) Right auricle shows resolution of erythema and redness after medical management.

## 3. Discussion

RES is a rare clinical entity characterized by recurrent episodes of unilateral or bilateral erythema, warmth, and burning pain of the external ear [1]. Despite increasing recognition in the literature, its underlying pathophysiology remains poorly understood [8]. Several mechanisms have been proposed and can be broadly categorized into central and peripheral mechanisms [7].

Central mechanisms primarily involve dysfunction of the brainstem trigeminoautonomic network. Goadsby and Lipton proposed the trigeminoautonomic circuit dysregulation theory, suggesting impaired regulation between trigeminal nerve activation and facial parasympathetic outflow. According to this hypothesis, stimulation of the trigeminal nerve by various triggers leads to abnormal parasympathetic responses, resulting in vasodilation and pain [4, 7, 9]. In addition, Raielli et al. proposed that activation of the trigeminovascular system leads to the release of vasoactive substances, resulting in vasodilation, erythema, and pain [4, 7, 10]. These central mechanisms are supported by the strong association between RES and headache disorders, particularly migraine.

Furthermore, peripheral mechanisms have been implicated, particularly in secondary forms of RES. Lance et al. suggested that antidromic discharge from the upper cervical nerve roots (C2‐C3), often related to cervical spine pathology, leads to the release of vasodilatory neuropeptides, resulting in auricular erythema and pain. TMJ dysfunction has also been proposed as a peripheral trigger. In this model, non‐noxious stimuli such as heat, touch, or chewing activate a local axon reflex, causing antidromic nerve discharges that result in neurogenic inflammation, vasodilation, and pain [3, 4, 7].

The diagnosis of RES remains primarily a diagnosis of exclusion. A broad differential diagnosis must be considered, including otitis externa, erysipelas, eczematous dermatologic conditions, relapsing polychondritis which preserve ear lobule, and other inflammatory or infectious causes of auricular erythema and pain [11, 12]. A comprehensive ENT examination is essential, supported by basic and targeted laboratory investigations. Evaluation for cervical spine and TMJ pathology is mandatory in suspected cases of secondary RES. Magnetic resonance imaging (MRI) of the brain and TMJ should be considered in patients with high clinical suspicion or associated neurological symptoms [5].

Our literature review of 45 articles comprising 100 reported cases of RES (Tables 1 and 2) indicated that the condition predominantly affected females (63%), with a mean age of 36.6 years. The left ear was most commonly involved (36%), followed by bilateral involvement (24%). Primary RES was the most frequent subtype (58%), followed by secondary and mixed forms (21% each). Headache disorders were present in 62% of the cases, with migraine accounting for 44%, further supporting the involvement of trigeminoautonomic and trigeminovascular mechanisms. Identifiable triggering factors were reported in 73% of the cases, reinforcing the contribution of both central and peripheral stimuli.

**TABLE 1:** Literature review of demographic data, clinic presentation, and characteristics of RES.

Variable	Frequency (*n*)	Percentages (%)
Sex		
Male	37	37
Female	63	63
Mean age	36.6 Y	
Max age	92 Y	
Min age	1.5 Y	
Affected ear		
Right ear	21	21
Left ear	36	36
Bilateral ear	24	24
Not mentioned	19	19
History of headache		
Yes	18	18
Yes‐Migraine	44	44
No	38	38
Secondary cause		
Yes	42	42
No	58	58
Triggering factors		
Yes	73	73
No trigger	13	13
Not mentioned	14	14
Relieving factors		
Yes	22	22
No	1	1
Not mentioned	77	77
Types		
Idiopathic	58	58
Secondary	21	21
Mixed	21	21

Abbreviation: RES, red ear syndrome.

**TABLE 2:** Literature review of case numbers, demographic data, clinic presentation, and characteristics of RES.

SN	Year	Author	CN	Age	Sex	Presentation of RES	Ear(s)	Headache	Secondary cause	Triggers	Relieving factor	Management
1	1996	Lance [3]	12									
			1	83	M	Right ear pain (burning sensation) and redness	R	N	Cervical arachnoiditis	Touching the ear	NM	Methysergide
			2	73	F	Left ear pain and warmth	L	N	Cervical spondylosis	Coughing, sneezing, or flexing her neck while turning to the left	NM	Indomethacin
			3	62	M	Left ear pain, redness, and warmth	L	Y	Cervical spondylosis	NM	NM	NM
			4	37	M	Left ear pain (burning sensation), redness, and warmth	L	N	Cervical spondylosis	Exertion	Cold pack or had a cold shower	Propranolol and C2‐C3 block
			5	33	F	Right ear pain (burning sensation) and redness	R	N	Traction injury of upper cervical roots	Neck movement and stress	NM	NM
			6	50	F	Left ear pain, redness, and swelling	L	Y‐M	Atypical glossopharyngeal and third cervical root neuralgia	Touch	NM	Flunarizine, C3 root block and C3 section
			7	35	M	Left ear pain and redness	L	N	Atypical glossopharyngeal and third cervical root neuralgia	Opening mouth, drinking or ear exposure to cold	NM	Surgical section of left glossopharyngeal nerve
			8	12	M	Bilateral ear pain and redness	B/L	N	TMJ dysfunction	Chewing or tooth grinding	NM	Dental plate
			9	42	F	Left ear pain (burning sensation) and redness	L	Y‐M	Atypical trigeminal neuralgia: Dysfunction	Menstrual period or any emotional stress	NM	Amitriptyline
			10	42	F	Left ear pain and redness	L	Y‐M	Thalamic syndrome with secondary migraines symptoms	NM	NM	Imipramine
			11	44	F	Right ear pain and redness	R	Y	—	heat	Ice pack to the ear	Supportive measures
			12	14	F	Right ear pain (burning sensation) and redness	R	Y	—	Touch, heat, hair brushing or chewing	NM	No treatment

2	1998	Dodick [13]	1	59	F	Left ear pain and redness	L	Y	Extra trigeminal EPH	Headache	NM	Indomethacin

3	1998	Boes et al. [14]	2									
			1	42	F	Left ear pain and redness	L	Y	CPH	NT	NM	Indomethacin
			2	49	F	Right ear pain	R	Y	CPH	NT	NM	Indomethacin

4	2000	Lee et al. [15]	1	9	M	Bilateral ear pain (burning sensation), redness, and swelling	B/L	Y	Neuropsychiatric SLE, vasculitic lesion of left MCA	NT	NM	Diazepam and methylprednisolone

5	2002	Raieli et al. [10]	8									
			1	10	M	Bilateral ear pain (burning sensation) and redness	B/L	Y‐M	—	NT	Ice pack to the ear	Nimodipine
			2	8	M	Ear pain and redness	NM	Y‐M	—	NT	NM	NM
			3	9	M	Left ear pain (burning sensation) and redness	L	Y‐M	—	Migraine attack	NM	NM
			4	12	F	Left ear pain (burning sensation) and redness	L	Y‐M	—	Migraine attack	NM	NM
			5	5	M	Bilateral ear pain (burning sensation) and redness	B/L	Y‐M	—	Migraine attack	NM	NM
			6	13	M	Right ear pain (burning sensation) and redness	R	Y‐M	—	Stress or migraine attack	NM	NM
			7	11	M	Right ear pain (burning sensation) and redness	R	Y‐M	—	Migraine attack	NM	Flunarizine
			8	36	F	Left ear pain (burning sensation) and redness	L	Y‐M	—	Migraine attack	NM	NM

6	2004	Donnet and Valade [16]	2									
			1	22	M	Bilateral ear pain (burning sensation) and redness	B/L	Y‐M	TAC	Migraine attack	NM	NM
			2	92	F	Left ear pain (burning sensation) and redness	L	N	TAC	NT	NM	NM

7	2004	Kumar and Swanson [17]	2									
			1	66	M	Left ear pain (burning sensation), redness, and warmth	L	N	Cervical root neuralgia (degenerative changes at C4 and C5 interspace with narrowing of the left C4 neural foramen)	Touch	NM	NM
			2	66	M	Right ear pain (burning sensation) and warmth	R	N	Cervical root neuralgia (spur at the C4 interspace on the right with narrowing of C4 neural foramen)	Head movement	NM	NM

8	2004	Ramirez and Kirsner [18]	1	53	M	Bilateral ear pain (burning sensation) and redness	B/L	N	Primary erythromelalgia	Heat or physical activity	Cooling	Multiple medication with no effect

9	2004	Evans and Lance [19]	1	54	F	Left ear pain (burning sensation) and redness	L	Y‐M	—	NM	NM	NM

10	2005	Al‐Din et al. [20]	18									
			1	42	F	Ear pain, redness, and hotness	NM	N	—	Change temperature	NM	Gabapentin
			2	58	F	Ear pain, redness, and hotness	NM	N	TMJ dysfunction	Laying down or TMJ touch	NM	Gabapentin
			3	51	F	Ear pain, redness, and hotness	NM	Y‐M	—	Change temperature	NM	Gabapentin
			4	39	F	Ear pain, redness, and hotness	NM	N	TMJ dysfunction	Laying down or TMJ touch	NM	Gabapentin
			5	49	F	Ear pain, redness, and hotness	NM	Y‐M	—	Change temperature	NM	Gabapentin
			6	45	F	Ear pain, redness, and hotness	NM	Y‐M	TMJ dysfunction	Laying down or TMJ touch	NM	Gabapentin
			7	37	F	Ear pain, redness, and hotness	NM	N	Pleomorphic adenoma of carotid body (ipsilateral) + TMJ dysfunction	Laying down or TMJ touch	NM	Gabapentin
			8	76	F	Ear pain, redness, and hotness	NM	N	TMJ dysfunction	Laying down or TMJ touch	NM	Gabapentin
			9	54	F	Ear pain, redness, and hotness	R	Y‐M	—	Change temperature or migraine attack	NM	Gabapentin
			10	57	F	Ear pain, redness, and hotness	NM	N	TMJ dysfunction	Laying down or TMJ touch	NM	Indomethacin
			11	29	F	Ear pain, redness, and hotness	L	Y‐M	—	Change temperature or migraine attack	NM	Gabapentin
			12	60	M	Ear pain, redness, and hotness	NM	N	—	Change temperature	NM	Gabapentin
			13	62	F	Ear pain, redness, and hotness	NM	Y‐M	—	NM	NM	NSAID
			14	36	F	Ear pain, redness, and hotness	NM	N	TAC	NM	NM	Gabapentin
			15	56	F	Ear pain, redness, and hotness	NM	Y‐M	—	NM	NM	Verapamil and gabapentin
			16	25	F	Ear pain, redness, and hotness	NM	N	TMJ dysfunction	NM	NM	Verapamil
			17	50	F	Ear pain, redness, and hotness	NM	N	—	NM	NM	Gabapentin
			18	25	F	Ear pain, redness, and hotness	NM	Y‐M	TMJ dysfunction	NM	NM	Gabapentin

11	2005	Arjona et al. [21]	5									
			1	70	F	Ear pain and redness	L	Y	Herpes zoster	Herpes zoster	NM	Famciclovir, prednisone and amitriptyline
			2	23	F	Ear pain and redness	R	Y	—	NM	NM	Amitriptyline
			3	41	M	Ear pain and redness	L	Y	Chiari I malformation	Sexual intercourse	NM	Amitriptyline and indomethacin
			4	26	F	Ear pain and redness	R	Y‐M	—	NM	NM	Amitriptyline and piroxicam
			5	48	F	Ear pain and redness	L	N	TMJ dysfunction	NM	NM	Amitriptyline

12	2007	Boulton et al. [22]	3									
			1	49	F	Left ear pain and redness	L	Y‐M	TMJ dysfunction, idiopathic congenital fusion of C1–C3 and enlargement of the cervical spinal canal	cold	Compression of ear	Gabapentin and ibuprofen
			2	66	F	Left ear pain and redness	L	N	—	—	Head movements	Ibuprofen
			3	58	F	Left ear pain and redness	L	Y‐M	TMJ dysfunction	Heat or touch	NM	Indomethacin

13	2009	Brill et al. [23]	1	7	M	Bilateral ear pain (burning sensation), redness, and swelling	B/L	Y	—	heat	cooling	Multiple medication with no effect

14	2009	Selekler et al. [24]	1	9	M	Bilateral ear pain (burning sensation) and redness	B/L	N	—	Touch	NM	Great auricular nerve block

15	2010	Creach et al. [25]	1	44	F	Right ear pain and redness	R	Y	C3 root and vertebral artery compression	Head movement or hard physical activity	NM	Neurovascular decompression of right vertebral artery

16	2011	Eismann et al. [12]	1	31	F	Right ear pain (burning sensation), redness, and swelling	R	Y‐M	—	Emotional stress, rubbing of the ear lobe or heat	cooling	Topical steroid

17	2012	Anesi et al. [26]	1	56	F	Bilateral ear pain, redness, and swelling	B/L	N	—	Ara‐C (anticancer drug)	NM	No intervention

18	2012	Ryan et al. [8]	1	23	F	Left ear pain (burning sensation), redness	L	N	—	NT	NM	Multiple medication with no effect

19	2013	Picco et al. [27]	1	10	M	Left ear pain (burning sensation) and redness	L	Y‐M	Cochleovestibular symptomatology	Heat	NM	Carbamazepine

20	2013	Lambru et al. [6]	1	69	F	Right ear pain (burning sensation) and redness	R	Y	SUNCT	Wind, touch, cleaning her teeth, eating, drinking, or brushing her hair	NM	Lamotrigine

21	2014	Lai et al. [28]	1	39	M	Left ear pain (burning sensation) and redness	B/L	N	TMJ dysfunction	TMJ movement or touch	NM	NSAID

22	2014	Chen et al. [11]	1	18	F	Bilateral ear pain (burning sensation), redness, and warmth	B/L	N	—	Warmth and movement, cleaning her teeth, eating, drinking, brushing her hair, touching, rubbing the ears or neck movements	Cold environment	Aspirin

23	2014	Chan and Ghosh [29]	1	22	F	Right ear pain (burning sensation), redness	R	Y‐M	—	Stress or orange juice	Avoidance of trigger	Supportive management

24	2014	Moitri et al. [30]	1	5	M	Bilateral ear pain (burning sensation), redness, and swelling	B/L	N	—	NT	NM	Cetirizine

25	2014	Uca and Kozak [31]	1	11	M	Right ear pain (burning sensation) and redness	R	Y‐M	—	Migraine attack	NM	Amitriptyline

26	2014	Flicinski et al. [32]	1	11	F	Left ear pain (burning sensation), redness, and swelling	L	Y‐M	—	Heat	—	Carbamazepine with no effect

27	2015	Lisotto et al. [33]	1	28	F	Left ear pain (burning sensation), redness	L	N	—	NM	NM	Pregabalin

28	2016	Velasco et al. [34]	1	26	F	Right ear pain (burning sensation) and redness	R	Y‐M	—	Migraine attack	Ice pack to the ear.	Topiramate

29	2016	Kalladka et al. [35]	1	41	M	Left ear pain (burning sensation) and redness and swelling	L	Y‐M	—	Migraine attack, hot temperature, or sun exposure	NM	Gabapentin

30	2017	Coleman and Ream [36]	1	10	M	Bilateral ear pain (burning sensation), redness, and swelling	B/L	Y‐M	—	Touch, heat or stress	Cooling	Verapamil

31	2017	Chan et al. [37]	1	35	M	Bilateral ear pain (burning sensation) and redness	B/L	Y	—	Hot temperature or alcohol intake	NM	Indomethacin

32	2018	Sahu et al. [38]	1	36	F	Bilateral ear erythema	B/L	N	—	Ara‐C (anticancer drug)	Stop the medication	Stop the medication

33	2018	La Mantia and Andaloro [39]	1	4	M	Left ear pain (burning sensation) and redness	L	Y‐M	—	Migraine attack	NM	Cetirizine

34	2018	de Amorim et al. [40]	6									
			1	22	F	Left ear pain, redness, and swelling	L	Y‐M	—	NT	NM	Sumatriptan, indomethacin, and oxygen
			2	82	F	Left ear pain and redness	L	Y	—	NT	NM	Steroids and onabotulinum toxin A
			3	38	F	Left ear pain and redness	L	Y‐M	—	stress	NM	Naproxen
			4	44	M	Right ear pain and redness	R	Y‐M	—	NT	NM	Multiple medication with no effect
			5	45	F	Right ear pain and redness	R	Y‐M	—	NT	NM	Multiple medication with no effect
			6	67	F	Left ear pain and redness	L	Y‐M	Bilateral reduction of the C3‐C4 foraminal amplitude	NT	NM	Amitriptyline with no effect

35	2019	Waseem et al. [41]	1	60	F	Bilateral ear erythema	B/L	N	—	Ara‐C(anticancer drug)	NM	Prednisone and oral diphenhydramine

36	2019	Cinats and Haber [42]	1	11	M	Bilateral ear pain (burning sensation), redness, and swelling	B/L	Y	—	Laying down on ears, tactile stimulation or stress	Ice pack to the ear	Gabapentin

37	2020	Brackenrich et al. [43]	1	57	M	Bilateral ear pain (burning sensation), redness, and swelling	B/L	N	Degenerative changes C4‐5	Sun or heat	Cooling	Paroxetine and aspirin

38	2020	Patel et al. [7]	3									
			1	23	F	Left ear pain and redness	L	N	—	Sunlight, wearing hairband, combing hair, lack of sleep, or travel postlunch	Ice pack to the ear and rest	Amitriptyline
			2	21	M	Left ear pain and redness	L	Y	—	Missing breakfast, sun, or traveling	Paracetamol and sleep	Flunarizine
			3	26	F	Bilateral ear pain (burning sensation) and redness	B/L	N	—	Eating sour food, strong perfume, getting emotional, or speaking a lie	Paracetamol and rest	No treatment

39	2020	Soares et al. [44]	1	57	F	Left ear pain (burning sensation) and redness	L	Y‐M	Tympanoplasty	Hot water or air	NM	Duloxetine

40	2021	D’Amico et al. [45]	3									
			1	11	M	Bilateral ears pain (burning sensation) and redness	B/L	Y‐M	—	Migraine attack	NM	Levosulpiride and valproic acid
			2	8	F	Bilateral ear pain and redness	B/L	N	—	Stress	Ice pack to the ear.	Magnesium supplement
			3	1.5	M	Right ear pain and redness	R	Y	TAC	NM	NM	No treatment

41	2022	Ye et al. [1]	1	12	M	Bilateral ear pain (burning sensation), redness, and warmth	B/L	N	Cervical spondylopathy	Warmth, movement, stress, excitement, or climbing stairs	Cold water, staying in cold room, and sitting up at night hours	Systemic itraconazole

42	2022	Sigdel and Pokhrel [46]	1	14	M	Bilateral ear pain (burning sensation) and redness	B/L	N	—	Summer season	No relieving factor	Indomethacin

43	2024	Callan et al. [47]	3									
			1	15	F	RES symptoms (not mentioned exactly the symptoms)	NM	Y‐M	—	Migraine attack	NM	Sumatriptan and ibuprofen
			2	47	F	Bilateral ear pain and reddening	B/L	Y‐M	—	Migraine attack	NM	Magnesium oxide and ubrogepant
			3	67	F	RES symptoms (not mentioned exactly the symptoms)	NM	Y‐M	—	Migraine attack	NM	Onabotulinum toxin A

44	2025	Ulgen Temel et al. [48]	1	16	F	Right ear pain (burning sensation) and redness	R	Y‐M	—	Migraine attack	NM	Indomethacin

45	2025	Sandakly et al. [49]	1	14	M	Left ear pain (burning sensation) and redness	L	N	—	Stress, rubbing, or touching the ear or heat	Ice pack to the ear.	Adalimumab, ibuprofen, a tricyclic antidepressant (TCA), and a calcium channel blocker

Abbreviations: B/L, bilateral; CPH, chronic paroxysmal hemicrania; EPH, episodic paroxysmal hemicrania; F, female; L, left; M, male; N, no; NM, not mentioned; NT, no trigger; R, right; SUNCT, short‐lasting unilateral neuralgiform headache attacks with conjunctival injection and tearing; TAC, trigeminal autonomic cephalalgias; TMJ, temporomandibular joint; Y, yes; Y‐M, Yes‐Migraine.

Several unusual presentations highlight the heterogeneity of RES. Arjona et al. reported cases of RES following herpes zoster infection attributed to injury of the upper cervical nerve roots and a case triggered by sexual activity in a patient with Chiari Type I malformation, likely due to transient compression of the cerebellar tonsils during exertion [21]. Picco et al. described a pediatric case characterized by burning ear pain associated with subjective tinnitus, transient hearing loss, auditory hallucinations, and vertigo, despite normal ENT and audiological evaluations [27]. Drug‐induced RES has been reported, particularly in association with cytarabine (Ara‐C), an antineoplastic agent used in hematological malignancies such as acute myeloid leukemia [26, 38, 41]. Additionally, Soares et al. described RES following tympanoplasty, hypothesizing that local neuronal injury during surgery triggered symptom onset, supported by rapid symptom relief with topical lidocaine and neuropathic pain medications [44].

No specific or universally effective treatment for RES has been established. Consequently, management strategies are largely guided by the proposed underlying pathophysiological mechanisms [4, 5]. Treatment of primary RES focuses on controlling the associated headache disorder, whereas the management of secondary RES targets the underlying causes. In our case, we started the patient on magnesium supplementation and indomethacin believing that it will target migraine and TACs. Magnesium supplementation is a good option for migraines, and indomethacin is an excellent option for TACs [50, 51].

In our literature review, management strategies demonstrated considerable variability, with therapies largely targeting the presumed underlying mechanisms rather than the syndrome itself. Pharmacological treatment was primarily dominated by neuropathic pain and migraine‐oriented therapies, with gabapentin being the most frequently reported medication, followed by indomethacin and amitriptyline. This observation supports the growing evidence suggesting that neuropathic mechanisms, central sensitization, trigeminovascular activation, and migraine‐related pathways may contribute significantly to RES pathogenesis. Gabapentin was commonly utilized in cases associated with neuropathic pain, cervical pathology, or temporomandibular disorders, whereas indomethacin appeared particularly useful in patients with overlapping TACs and paroxysmal headache syndromes. Similarly, the frequent use of migraine prophylactic therapies including flunarizine, verapamil, topiramate, magnesium supplementation, triptan‐based therapies, and calcitonin gene–related peptide (CGRP)–related agents further reinforces the close association between RES and migraine‐spectrum disorders. Nonpharmacological interventions were similarly heterogeneous and predominantly focused on trigger avoidance and symptomatic relief. Cooling measures, including ice packs, cold environments, and cold‐water application, were frequently reported as effective acute symptomatic strategies, likely due to their vasoconstrictive effects and modulation of neurovascular activation. Furthermore, several cases required more targeted interventions directed toward identifiable secondary causes, including dental appliances for temporomandibular disorders, medication discontinuation for drug‐induced cases, nerve blocks, cervical root interventions, neurovascular decompression procedures, and other surgical approaches for structural pathology. More recently, advanced therapies such as onabotulinum toxin A have emerged as potential options for refractory cases although evidence remains limited to isolated reports.

Therapeutic outcomes remain highly variable. Many reported cases require multiple treatment trials with limited benefit, whereas others demonstrate spontaneous resolution. In selected cases of secondary RES, surgical intervention has been necessary as part of definitive management [3, 25].

A limitation in this case is that RES is a rare disease, and the diagnosis is mainly established by excluding other diseases with similar clinical presentations. Furthermore, there is currently no clear evidence supporting racial or ethnic predilection in RES. However, this approach also represents a strength, as comprehensive evaluation and careful exclusion of alternative conditions further strengthened and supported the diagnosis of RES.

## 4. Conclusion

RES is a rare and often underrecognized condition that should be considered in patients presenting with recurrent auricular erythema and burning pain, particularly in those with migraine. This case highlights the importance of a thorough evaluation and exclusion of alternative diagnoses to avoid misdiagnosis and mismanagement. Early recognition of RES may facilitate appropriate treatment, reduce unnecessary interventions, and improve patient outcomes.

## Author Contributions

Zaki Alwatban: manuscript writing literature and review analysis of the reviewed article.

Wasan Alharbi: manuscript writing and literature review.

Muneera Al Khalifa: manuscript review and analysis result review.

Mohammed Al Shehabi: manuscript review and analysis result review.

## Funding

The authors did not receive support from any organization for the submitted work.

## Disclosure

All authors have read and approved the final manuscript.

## Ethics Statement

The study was approved by the Royal Medical Services Ethical and Medical Committee, Bahrain (approval number: RMS‐MH/IRB/2026‐1114).

## Consent

Written informed consent was obtained from the patient.

Consent was obtained from patient for publishing the case without disclose any confidential information.

## Conflicts of Interest

The authors declare no conflicts of interest.

## Patient Perspective

The patient experienced ear pain and redness, mainly triggered by migraine attacks, exposure to hot weather, and ear rubbing. The patient was dissatisfied due to multiple clinic visits without any improvement. However, after the diagnosis of RES was established and appropriate management was initiated, the patient was satisfied with the treatment outcomes.

## Data Availability

My manuscript has no associated data or the data will not be deposited.
